# Supplementary material for: MetaRibo-Seq measures translation in microbiomes
Source: Nat Commun. 2020 Jun 29;11:3268. doi: 10.1038/s41467-020-17081-z (PMC7324362; doi:10.1038/s41467-020-17081-z)
Supplement: Supplementary file 10 — Supplementary Data 7 [file 41467_2020_17081_MOESM10_ESM.zip › File2/Confidence_VeryHigh_Taxonomy/196756_out.krona.html]

Javascript must be enabled to view this page.

members
magnitude
magnitudeUnassigned
count
unassigned
taxon
rank

196756\_out

4

2
4
superkingdom

1239
4
phylum

186801
4
class

4
186802
order

family
541000
1

1
1263
genus

SRS049995\_contig\_number\_contig-100\_18109.98351

family
186803
3

33042
1
genus


SRS147346\_contig\_number\_43658
410072
1
species

genus
1
572511


SRS148511\_contig\_number\_17827
33038
1
species

genus
1
841


SRS893259\_contig\_number\_4776
1
360807
species
